# Acute and Subacute Toxicity of *Rhamnus prinoides* Leaves on Histopathology of Liver, Kidney, and Brain Tissues, and Biochemical Profile of Rats

**DOI:** 10.1155/2023/3105615

**Published:** 2023-01-18

**Authors:** Melese Shenkut Abebe

**Affiliations:** Department of Anatomy, School of Medicine, College of Medicine and Health Science, Wollo University, Dessie, Ethiopia

## Abstract

*Rhamnus prinoides* is used as a traditional medicinal plant to treat pneumonia, sprain, gonorrhea, rheumatism, and ringworm infections as well as for the preparation of local beverages in Ethiopia. It has a widespread antioxidant, antimalarial, antimicrobial, wound healing, and anti-inflammatory activities. These activities are due to the presence of alkaloids, steroids, triterpenes, tannins, flavonoids, flavones, phenols, and glycosides. This study aimed to investigate acute and subacute toxicity of *R. prinoides* leaves on histopathology of the liver, kidney, and brain tissues, and biochemical profiles of rats. For the acute toxicity study, female rats were treated with *R. prinoides* at a dose of 5000 mg/kg body weight and followed-up for 14 days. In the subacute toxicity study, four groups of rats were used. The first three groups, respectively, received 250, 500, and 1000 mg/kg body weight of *R. prinoides* extract and the fourth group was a control group. Signs of toxicity, food intake, and weight was recorded. At necropsy, organ weight measurement and macroscopic and microscopic evaluations of the liver, kidney, and brain were carried out. Different clinical chemistry profiles of rats were also measured. Single-dose oral administration of *R. prinoides* extract at 5000 mg/kg produced no mortality indicating the LD_50_ is greater than 5000 mg/kg body weight. A four week administration of *R. prinoides* extract did not bring deleterious outcomes on the food consumption and weight gain of rats. Moreover, gross examination, histopathological evaluation, and weight measurement conducted on the liver, kidney, and brain did not reveal treatment related changes. The biochemical analysis showed no significant difference between the treatment and control groups. Consumption of *R. prinoides* leaf for 4 weeks might not have a toxic effect in rats. However, further investigations upon long-term administration should be conducted to have a wider safety margin.

## 1. Introduction

Consumption of herbal products has a remarkable contribution to the prevention and treatment of various diseases. Plant products have been used for a long time because they are a natural source of therapeutic agents [1]. *R. prinoides* is a bushy evergreen tree that may reach up to 9 m in height [2, 3]. It is a widespread plant species in East, Central, and South African countries [2, 4, 5]. In Ethiopia, there is wide cultivation of *R*. *prinoides* in Amhara region, North Shoa zone: Antsekuyana-Gemza, Efratana-Gidim, and Gina Ager [5].

In Ethiopia, *R*. *prinoides* is routinely used to prepare traditional alcoholic beverages called Tella and Tej. Research finding indicates that *R. prinoides* can replace the standard commercial hops used for beer preparation. Hops used during beer production provide flavor, aroma, and bittering tastes for the beer [6]. During the preparation of Tella, *R. prinoides* is found to inhibit bacterial growth, which helps to expand the shelf-life of Tella [7].

The leaf of *R. prinoides* is traditionally used for the treatment of diseases such as stomachache, joint pain, fever, diarrhea, common cold, malaria, body weakness, loss of appetite, pneumonia, sprain, gonorrhea, rheumatism, and ringworm infections [2, 4]. A research finding showed that different parts of the plant have been reported with tremendous antioxidant, antimalarial, antimicrobial, wound healing, and anti-inflammatory activities [8].

The phytochemical screening of *R. prinoides* leaf showed that it has alkaloids, steroids, triterpenes, tannins, flavonoids, flavones, phenols, glycosides, anthraquinones, and resins. Nutritional analysis of *R. prinoides* also indicated that it has high carbohydrate (70.5%), fiber (25.6%), crude protein (8.5%), and lipid (3.5%) contents [8].

Many research findings have shown that plenty of medicinal plants have toxic effects on animals and humans. Their toxicity ranges from altering cellular and biochemical components of blood to the histopathological changes of various internal organs [9]. Continuous and growing demand for herbal therapies has invigorated the quest for validating the potency and harmlessness of medicinal plants [10]. Despite widespread consumption of *R*. *prinoides* for the preparation of beverages and medicinal purposes, the safety of this plant has not been scientifically evaluated yet. Thus, the adverse effects of *R*. *prinoides* on various blood parameters and histopathology of the liver, kidney, and other vital organs are not known. This toxicological study was carried out in an attempt to get safe, socially acceptable, and cheap antipathogenic agents of plant origin as well as to prevent the local community who liberally used the plant for medicinal and nutritional purposes. Therefore, the present study is concerned to assess the toxic effect of *R*. *prinoides* on the tissue of the liver, kidney, and brain, and its effect on the biochemical profile in rats.

## 2. Materials and Methods

### 2.1. Plant Collection and Extraction

Fresh leaves of *R. prinoides* were gathered from local farmers around Addis Ababa, Ethiopia. The plant was identified and authenticated by an expert taxonomist and a voucher specimen (MS 002) was deposited. The overall plant extraction was performed following the protocol described by Abebe et al. [11, 12]. The leaves of the *R. prinoides* were cleaned, dried at ambient temperature, and powdered. We used 70% ethanol to macerate the powder and the mixture was agitated by an orbital shaker for 24 hours, which came after by filtration using Whatman paper (No 1, 18 cm diameter) and concentration with a rotatory evaporator (Büchi Rota Vapor R-205, Switzerland) at 40°C. The concentrate was further dried by a hot water bath at 45°C. Lastly, the endmost extract was stowed in the refrigerator.

### 2.2. Experimental Animals

Rats were used for this research. Male and female rats, 2-3 months of age and weighing 200–220 g, were obtained from colonies in the animal house of the Department of Pharmacology, Addis Ababa University. The rats were acculturated to the lab setup for seven days before extract administration. The test animals were fed with a standard commercial diet and water, maintained at a standard laboratory temperature, relative humidity, and 12 hr light/night cycle until the end of the experiment. All experimental methods including animal handling were performed in harmony with internationally accepted guidelines and following the approval by the ethical review board of Wollo University.

### 2.3. Experimental Design

#### 2.3.1. Acute Toxicity

In the preliminary acute toxicity study, we followed Organization for Economic Cooperation and Development (OECD) guideline Test No 423 [13]. Three female Wistar rats were used as the treatment group while the other three rats were set as a control group. Before administration of the extract, rats fasted overnight from food but not water. Fasted body weight was measured and doses were calculated based on this weight. Since the leaves of *R. prinoides* are extensively consumed orally as a component of a local beverage called Tella and Tej, we have used a higher starting dose. The treatment group received *R. prinoides* leaf extract at a dose of 5000 mg/kg body weight whereas the control group was given distilled water. After administration of the extract, food was withheld for 4 hours. Close follow-up was performed for the first four hours then every hour in the first 24 hours and every day for the next 14 days. During the follow-up period, all signs of toxicity were recorded. At the end of the 14^th^ day, rats were killed with an overdose of pentobarbital and dissected. Gross assessments of visceral organs including the liver and kidney were performed. Any morphological change was recorded. In addition, the weight of the liver and kidney was recorded.

#### 2.3.2. Subacute Toxicity Study

Subacute toxicity was conducted following the protocols stated in OCDE guideline Test No. 407 [14]. Based on the recommendation by OECD Test No 407, forty Wistar rats (5 male and 5 female/group) were employed for subacute toxicity testing. They were divided into four groups. Group I–III obtained 250, 500, and 1000 mg/kg body weight of *R. prinoides* extract and group IV, control, received distilled water. Doses were selected based on our preliminary study results. Administration of the test substance was given by intragastric tube, on daily basis for four weeks (28 days). In pre- and post-treatment periods, all rats were inspected for the existence of any sign of toxicity. Behavioral and physical signs of toxicity were recorded. In addition, daily feed consumption was measured and the body weight of rats was taken at the inception of treatment, weekly afterwards, and at the termination of the treatment schedule.

### 2.4. Organ Weight and Macroscopic Examination

At the culmination of the treatment schedule, rats were killed by intraperitoneal (IP) injection of pentobarbital. Major visceral organs mainly the liver, kidney, and brain were inspected for any gross morphological abnormalities. The weight of the aforementioned organs was also measured.

### 2.5. Determination of Biochemical Parameters

While the rats were under anesthesia, fasted blood was obtained via cardiac puncture. The blood was kept in a test tube with no anticoagulant. Following centrifugation for 10 minutes, by an electrical centrifuge, at 3500 rpm, serum was collected by micropipette. Biochemical parameters referring liver and kidney functions, alanine aminotransferase (ALT), aspartate aminotransferase (AST), alkaline phosphatase (ALP), urea, creatinine, total protein, albumin, glucose, and total cholesterol, were recorded by an automated clinical chemistry analyzer.

### 2.6. Histopathological Investigation

A 3 mm tissue sample from the liver, kidney, and brain was taken and preserved in 10% formalin for a purpose of fixation. For the routine tissue processing steps: dehydration, clearing, embedding, mounting, and staining, we followed the methods discussed by Abebe et al. [15].

### 2.7. Statistical Analysis

We have used the statistical package for social science (SPSS) version 24 for analyzing the data. The variation between treatment and control groups was identified by employing a one-way analysis of variance (ANOVA) followed by Turkey and Dunnett post-Hoc tests. Results are presented as mean ± standard error of the mean (SE). The statistical level of significance was set as *p* value <0.05.

## 3. Results and Discussion

### 3.1. Acute Toxicity Study

#### 3.1.1. Clinical Observation

Following a single-dose oral administration of *R. prinoides* at 5000 mg/kg body weight, within four hours of follow-up, some signs of toxicity such as piloerection, reduced response to touch, and decreased physical activity were observed. However, these signs were gradually decreased and finally disappeared in the next 24 hours of follow-up. No other signs of severe toxicity or death were recorded in the 14 days of follow-up. By this, it may possible to state that the LD_50_ of *R. prinoides* leaf is greater than 5000 mg/kg body weight.

#### 3.1.2. Morphology and Organ Weight

**Figure 1:**
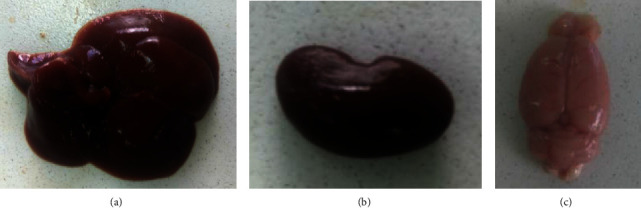
Pictures showing the normal liver (a), kidney (b), and brain (c) of rat treated with 1000 mg/kg body weight of *R. prinoides* extract.

No abnormal morphological change in the color, texture, and weight of liver and kidney was recorded (Figure 1). Weight gain was not significantly affected by treatment with a single dose of *R. prinoides* extract at 5000 mg/kg body weight compared with the control group (Table 1).

### 3.2. Subacute Toxicity Study Findings

#### 3.2.1. Clinical Observation Result

Following four weeks of administration of *R. prinoides* leaf extract to rats, no sign of toxicity in the behavior or general appearance was observed in any of the treatment groups. In addition, no mortality was recorded all around the treatment period.

### 3.3. Effect on Food Intake and Weight Gain

Following 28 days of *R. prinoides* extract administration, the food intake of treated male and female rats was not significantly different from the control groups. Although, the difference is not significant, the weight gain of rats treated with *R. prinoides* leaf extract was higher than the control group (Table 2). This finding might support the nutritional value of *R. prinoides*. Previous research [8] reported that *R. prinoides* has a high content of carbohydrate (70.5%), fiber (25.6%), and protein (8.5%), which shows its nutritional significance. In addition, consumption of *R. prinoides* leaves did not produce a loss of appetite or affect the normal growth of rats. It may be due to the absence of such effects that the local community, in many areas of Ethiopia, liberally used *R. prinoides* to brew traditional drinks such as Tella and Tej [16].

### 3.4. Gross Examination and Weight of Organs

Macroscopic examination conducted on the liver, kidney, and brain tissues did not reveal any gross structural alterations (Figure 1). In toxicological studies, evaluating organs weight is one of the integral components of the investigation. Multiple internationally recognized guidelines recommended measuring the weight of vital organs such as the liver, kidney, heart, brain, and others [14, 17]. In the current work, the absolute weight of the liver, kidney, and brain did not significantly vary between *R. prinoides* treated and control group (Table 3) indicating the test plant has no hurtful impact on the weight of the aforementioned organs.

### 3.5. Effects on the Biochemical Profile of Rats

The results of clinical chemistry paraments evaluation are presented in Tables 4 and 5. The liver is one of the most principal organs that prevents our body from toxic insults of ingested material. Changes in the liver function tests, usually due to liver cell impairment, has been communicated as a sign of hepatotoxicity [18]. In our study, any of the liver function tests measured, in both male and female rats, were not affected by the treatment of *R. prinoides*. Another organ that can be affected by toxicants entering into the systemic circulation is the kidney. The kidney functions in blood filtration, urine concentration, and metabolic activation of exogenous chemicals [19]. The value of kidney function tests that we have recorded did not show significant variation between *R. prinoides* treated and control groups. Overall, short-term administration of ethanol leaf extract of *R. prinoides* to male and female rats might not have a statistically significant deleterious effect on the functions of liver and kidney.

### 3.6. Effects on the Histopathology of Liver, Kidneys, and Brain

**Figure 2:**
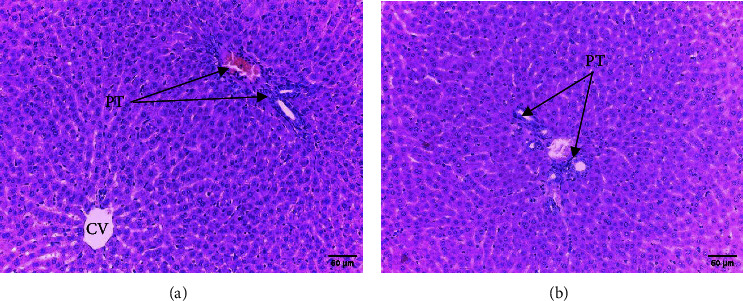
Photomicrograph showing normal rat liver, section (a) taken from rats in group III (1000 mg/kg), section (b) taken from rats in group IV (control); PT: portal triad, CV: central vein; H and E stain, and 200x magnification.

**Figure 3:**
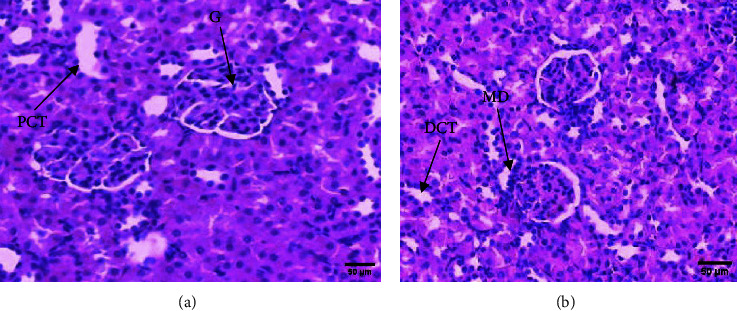
Photomicrograph showing normal rat kidney, section (a) taken from rats in group III (1000 mg/kg), section (b) taken from rats in group IV (control); G glomerulus, PCT: proximal convoluted tubule, DCT: distal convoluted tubule, MD: macula densa cells; H and E stain, and 200x magnification.

**Figure 4:**
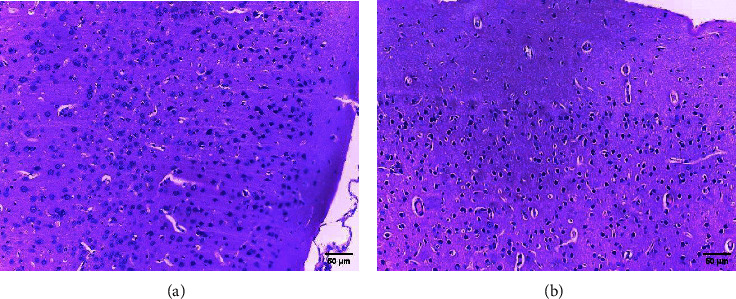
Photomicrograph showing normal rat brain (cerebrum), section (a) taken from rats in group III (1000 mg/kg), section (b) taken from rats in group IV (control); H and E stain, 200x magnification.

**Figure 5:**
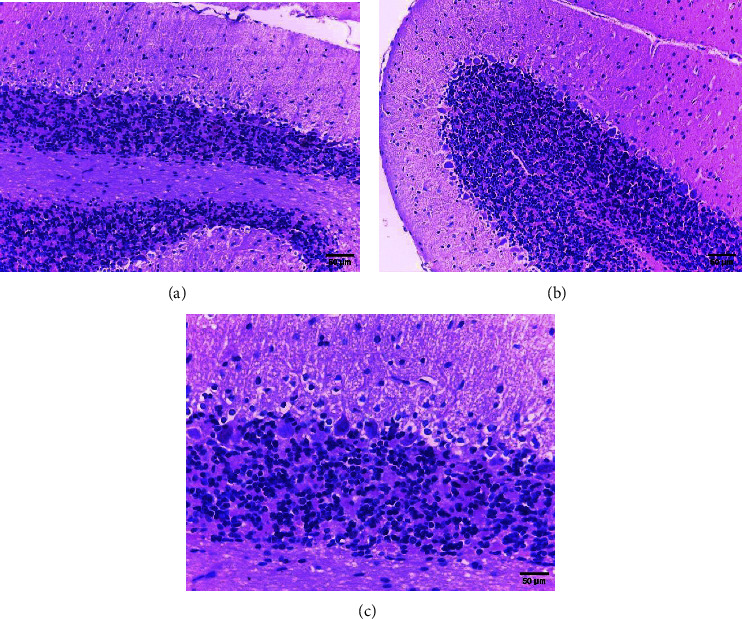
Photomicrograph showing normal rat brain (cerebellum), section (a and c) taken from rats in group III (1000 mg/kg), section (b) taken from rats in group IV (control); H and E stain, 200x (a and b), and 400x (c) magnification.

In safety assessment of medicinal plants, histopathological evaluations of organs provide informative evidence regarding the effects of a test substance on their microscopic structures [20, 21]. Administration of ethanol extract of *R. prinoides* leaves for four weeks did not reveal a significant structural change in hematoxylin and eosin (H & E) stained tissue of the liver. The liver's microscopic structures: the porta hepatis, biliary tract, hepatocytes, and microvasculature were normal (Figure 2). In addition, microscopic structures of the kidney were not affected by treatment with *R. prinoides* leaves extract up to 1000 mg/kg dose. The glomerular capillaries with its capsule, the afferent and efferent arterioles, and the kidney tubes did not show treatment related changes in all doses (Figure 3). Similarly, neurons and supporting cells of different regions of the brain were not affected by the plant extract (Figures 4 and 5). In the current investigation, the absence of microscopic change is consistent with the biochemical parameters measured. This indicates that the use of *R. prinoides* leaves for a short duration might have no noxious effect.

## 4. Conclusions

Based on the findings of the current investigation, administration of 70% ethanol extract of *R. prinoides* leaves at 5000 mg/kg did not produce mortality indicating the LD_50_ of *R. prinoides* is greater than 5000 mg/kg body weight. Consumption of *R. prinoides* leaf for a short duration (4 weeks) might not affect the appetite or normal growth of rats. Moreover, the function and microscopic structures of the liver, kidney, and brain may not be affected by short-term consumption of *R. prinoides* leaves. However, further investigations upon long-term administration of *R. prinoides* should be conducted to have a wider safety margin of the current test plant.

## Figures and Tables

**Table 1:** Weight gain and organ weight of rats: acute toxicity study.

Group	Weight gain	Organ weight	
		Liver	Kidney
5000 mg/kg	33.3 ± 0.33	8.5 ± 0.15	1.53 ± 0.06
Control	31.0 ± 2.31	8.4 ± 0.12	1.51 ± 0.01

Results are expressed as mean ± SE; one-way ANOVA.

**Table 2:** Food intake and weight gain of rats treated with leaf extract of *R. prinoides*.

Rats	Food intake and weight gain	Group			
		Group I	Group II	Group III	Group IV
Male	Food intake (g/day) *n* = 5	110.2 ± 1.8	111.2 ± 2.4	113.8 ± 3.3	116.0 ± 2.9
	Weight gain/rat (g)	82.3 ± 4.6	98.0 ± 1.7	99.7 ± 1.7	86.3 ± 5.2

Female	Food intake (g/day) *n* = 5	88.8 ± 2.4	80.8 ± 2.3	84.1 ± 3.2	78.6 ± 2.2
	Weight gain/rat (g)	30.0 ± 5.0	34.7 ± 6.7	32.0 ± 2.1	23.3 ± 1.5

Results are expressed as mean ± SE; one-way ANOVA. Group I–III, respectively, received 250, 500, and 1000 mg/kg of *R. prinoides* extract and group IV: control.

**Table 3:** Organ weight of rats treated with leaf extract of *R. prinoides*.

Absolute organ weight (g)		Group			
		Group I	Group II	Group III	Group IV
Male	Liver	8.92 ± 0.3	8.66 ± 0.2	9.01 ± 0.3	8.72 ± 0.2
	Kidney	0.92 ± 0.05	0.91 ± 0.03	1.10 ± 0.08	0.96 ± 0.06
	Brain	1.92 ± 0.03	1.89 ± 0.02	1.92 ± 0.04	1.98 ± 0.04

Female	Liver	6.62 ± 0.2	6.51 ± 0.2	6.93 ± 0.3	7.11 ± 0.4
	Kidney	0.67 ± 0.02	0.71 ± 0.01	0.76 ± 0.04	0.69 ± 0.01
	Brain	1.85 ± 0.01	1.88 ± 0.08	1.77 ± 0.04	1.83 ± 0.02

Results are expressed as mean ± SE; one-way ANOVA. Group I–III, respectively, received 250, 500, and 1000 mg/kg of *R. prinoides* extract and group IV: control.

**Table 4:** Biochemical profile of male rats treated with leaf extract of *R. prinoides*.

Tests	Group			
	Group I	Group II	Group III	Group IV
ALT (U/L)	40.01 ± 4.7	46.33 ± 5.2	55.01 ± 93.1	56.52 ± 3.5
AST (U/L)	193.22 ± 49.8	191.21 ± 7.8	216.82 ± 28.9	216.41 ± 23.5
ALP (U/L)	143.32 ± 24.3	154.01 ± 20.5	146.72 ± 12.2	162.74 ± 25.8
Urea (mg/dL)	43.05 ± 2.3	45.34 ± 2.6	43.86 ± 2.6	46.91 ± 1.9
Creatinine (mg/dL)	0.29 ± 0.06	0.33 ± 0.00	0.30 ± 0.01	0.31 ± 0.04
Albumin (g/dL)	4.49 ± 0.03	4.31 ± 0.04	4.55 ± 0.08	4.42 ± 0.09
Total protein (g/dL)	6.43 ± 0.28	6.07 ± 0.06	6.26 ± 0.09	6.26 ± 0.17
Total cholesterol (mg/dL)	51.42 ± 5.2	40.13 ± 1.2	40.15 ± 1.2	46.91 ± 1.7
Glucose (mg/dL)	121.33 ± 6.4	107.51 ± 19.3	80.54 ± 20.0	80.42 ± 6.6

Results are expressed as mean ± SE; one-way ANOVA. ALT: alanine aminotransferase, AST: aspartate aminotransferase, ALP: alkaline phosphatase. Group I–III, respectively, received 250, 500, and 1000 mg/kg of *R. prinoides* extract and group IV: control.

**Table 5:** Biochemical profile of female rats treated with leaf extract of *R. prinoides*.

Tests	Groups			
	Group I	Group II	Group III	Group IV
ALT (U/L)	50.32 ± 2.5	48.51 ± 1.8	44.53 ± 0.8	49.51 ± 3.4
AST (U/L)	181.42 ± 28.7	183.70 ± 2.8	201.71 ± 4.9	194.90 ± 9.3
ALP (U/L)	89.01 ± 10.0	84.02 ± 6.7	85.71 ± 4.1	75.74 ± 9.8
Urea (mg/dL)	45.10 ± 2.6	45.81 ± 2.3	40.74 ± 0.7	40.71 ± 2.1
Creatinine (mg/dL)	0.35 ± 0.03	0.38 ± 0.02	0.31 ± 0.04	0.32 ± 0.03
Albumin (g/dL)	4.39 ± 0.15	4.33 ± 0.07	4.55 ± 0.08	4.37 ± 0.09
Total protein (g/dL)	6.43 ± 0.12	6.21 ± 0.02	6.43 ± 0.14	6.28 ± 0.15
Total cholesterol (mg/dL)	65.51 ± 0.67	68.21 ± 8.4	66.62 ± 3.6	65.94 ± 3.9
Glucose (mg/dL)	103.22 ± 2.6	101.31 ± 2.9	93.02 ± 1.2	93.10 ± 3.5

Results are expressed as mean ± SE; one-way ANOVA. ALT: alanine aminotransferase, AST: aspartate aminotransferase, ALP: alkaline phosphatase. Group I–III, respectively, received 250, 500, and 1000 mg/kg of *R. prinoides* extract and group IV: control.

## Data Availability

All data are included in the manuscript.

## Abbreviations

ALP: Alkaline phosphatase
ALT: Alanine aminotransferase
ANOVA: Analysis of variance
AST: Aspartate aminotransferase
H & E: Hematoxylin and eosin
OECD: Organization for economic co-operation and development
SEM: Standard error of mean
SPSS: Statistical package for social science.
